# A Comparative Analysis of GISTs and Schwannomas in the Sigmoid Colon: Case Report and Review of the Management Strategies

**DOI:** 10.3390/jcm14113831

**Published:** 2025-05-29

**Authors:** George Ionut Golea, Radu Alexandru Ilies, Stefana Dascalescu, Dragos Stefan Morariu, Ioan Catalin Vlad

**Affiliations:** 1 Surgery 1, Cluj County Emergency Clinical Hospital, 400006 Cluj-Napoca, Romania; george.ionu.golea@elearn.umfcluj.ro; 2 Faculty of Medicine, Iuliu Hatieganu University of Medicine and Pharmacy, 400012 Cluj-Napoca, Romania; 3 Medical Oncology, Oncology Institute Prof. Dr. Ion Chiricuta, 400015 Cluj-Napoca, Romania; stefana.dascalescu@elearn.umfcluj.ro; 4 Surgical Oncology, Oncology Institute Prof. Dr. Ion Chiricuta, 400015 Cluj-Napoca, Romania; dragosstefanmorariu@gmail.com (D.S.M.); catalinvlad@elearn.umfcluj.ro (I.C.V.); 5 Surgical Oncology and Gynecologic Oncology, Iuliu Hatieganu University of Medicine and Pharmacy, 400012 Cluj-Napoca, Romania

**Keywords:** colorectal tumors, gastrointestinal stromal tumor, immunohistochemistry, mesenchymal tumors, schwannoma

## Abstract

**Background/Objectives:** Mesenchymal tumors of the gastrointestinal tract are rare and can pose significant diagnostic challenges, particularly when located in atypical sites such as the sigmoid colon. Gastrointestinal stromal tumors (GISTs) are often the primary consideration based on imaging findings; however, other spindle cell neoplasms, such as schwannomas, must also be considered. We present a case of a sigmoid colon schwannoma initially suspected to be a GIST and provide a literature review on the diagnostic and therapeutic challenges associated with these tumors. **Methods**: A literature review based on articles from 2015 to 2024 was conducted to identify cases of mesenchymal tumors of the colon misdiagnosed as GISTs. The review focused on the role of imaging, endoscopic biopsy, and immunohistochemistry in differentiating these neoplasms. Additionally, treatment approaches, including surgical resection versus targeted therapy, were assessed. **Results**: The literature review revealed that GISTs and schwannomas share overlapping imaging characteristics, including submucosal location, hyperintensity on T2-weighted MRI, and contrast enhancement. However, immunohistochemical markers remain the gold standard for differentiation. Studies also highlighted the increasing role of minimally invasive diagnostic techniques, such as fine-needle aspiration and molecular profiling, in achieving a definitive preoperative diagnosis. Unlike GISTs, which often require adjuvant therapy with tyrosine kinase inhibitors, schwannomas are typically treated with surgical excision alone, with a low risk of recurrence. **Conclusions**: Current evidence supports a multimodal diagnostic approach combining imaging, biopsy, and immunohistochemistry to accurately classify mesenchymal tumors of the colon. While imaging can suggest a probable diagnosis, histopathological confirmation is essential before initiating targeted therapy.

## 1. Introduction

### 1.1. Colorectal Gastrointestinal Stromal Tumors

Gastrointestinal stromal tumors (GISTs) are the most common mesenchymal neoplasms of the gastrointestinal tract, with an estimated incidence of 10–20 cases per million people per year. Although the stomach and small intestine are the predominant sites of origin, GISTs can also arise in the colon, particularly the sigmoid region, where they are considerably rarer and present unique diagnostic and therapeutic challenges [1,2,3]. These tumors present a broad spectrum of clinical manifestations, ranging from incidental findings during routine investigations to acute manifestations characterized by gastrointestinal bleeding, perforation, or even presentations that mimic other pelvic pathologies [1,2,4].

The diagnosis of GISTs typically relies on a multimodal approach. Advanced imaging modalities, including magnetic resonance imaging (MRI) and positron emission tomography-computed tomography (PET-CT), play a crucial role in delineating lesion morphology, highlighting features such as T2 hyperintensity, restricted diffusion, and contrast enhancement, and in assessing the extent of disease and potential metastatic spread [1,2,5]. Endoscopic techniques, such as colonoscopy and endoscopic ultrasound-guided fine-needle aspiration (EUS-FNA), further facilitate direct visualization and tissue sampling, enabling histopathological confirmation through the identification of characteristic spindle cell morphology and immunohistochemical positivity for markers such as CD117, DOG1, and CD34 [3,6,7,8].

The literature reveals a diverse range of clinical presentations and outcomes associated with sigmoid GIST. For example, several case reports have documented presentations ranging from subtle, nonspecific symptoms in elderly patients [1] to life-threatening hemorrhage in younger individuals [2]. In some cases, the lesions have been misinterpreted as other neoplastic processes (such as ovarian tumors or peripheral nerve sheath tumors), underscoring the need for careful differential diagnosis [7,9,10]. In addition, new diagnostic modalities, including colonic capsule endoscopy, have emerged as valuable adjuncts, particularly in cases where traditional colonoscopy is inconclusive [11]. Rare presentations, such as diffuse infiltrating tumors causing spontaneous perforation or pneumoretroperitoneum, have also been reported, highlighting the heterogeneous nature of this pathology [12,13,14,15].

Therapeutic strategies for sigmoid GISTs predominantly focus on complete surgical resection (R0 resection), which remains the cornerstone of curative treatment. The role of neoadjuvant and adjuvant targeted therapies—particularly imatinib mesylate—has been increasingly recognized, especially in cases of locally advanced disease or when recurrence is a concern [16]. In addition, hybrid lesions and atypical presentations, such as sporadic segmental interstitial cells of Cajal hyperplasia or gastrointestinal autonomic nerve (GAN) tumors, further expand the spectrum of GIST-related pathology and require precise molecular characterization for optimal management [16,17]. Reports of metastatic involvement, as well as series summarizing clinical outcomes, emphasize the importance of long-term follow-up and multidisciplinary management in these patients [18,19,20].

### 1.2. Colorectal Schwannomas

Colorectal schwannomas are rare, usually benign, mesenchymal tumors that are often discovered incidentally during screening colonoscopy [21]. About 2–6% of all cases arise in the gastrointestinal tract, with the small and large intestines being involved in about 12% of these occurrences [22]. These tumors can present with a variety of clinical symptoms ranging from asymptomatic to gastrointestinal complaints such as abdominal pain, rectal bleeding, and constipation [22]. Due to their submucosal location, they can be difficult to diagnose preoperatively, often requiring histopathological confirmation by immunohistochemistry with positivity for S100 protein [21]. Atypical and hybrid forms of colorectal schwannomas also exist. Hybrid schwannoma-perineuroma tumors, which co-express both Schwann and perineurial markers, such as S100 and CD34, have been identified in some cases, suggesting that they may represent a distinct subclass of these lesions [23]. In addition, some schwannomas present rare histologic variants, such as microcystic or reticular forms, which require differentiation from other potential colorectal pathologies, such as GISTs. The primary treatment for colorectal schwannomas is surgical resection, which is generally curative, especially when performed with clear margins. Occasionally, complications arise, such as misdiagnosis or co-occurrence with other malignancies, emphasizing the need for careful preoperative imaging and long-term follow-up in certain patients. Diagnosis involves both imaging features and immunohistochemical studies, with S100 positivity being a key marker for this tumor type [21,24,25].

Colorectal schwannomas are rare mesenchymal tumors that are usually benign and often discovered incidentally during routine screening or evaluation for nonspecific gastrointestinal conditions. Their generally indolent behavior and lack of aggressive features distinguish them from other mesenchymal neoplasms of the colon [21,22,26].

Preoperative diagnosis is challenging due to their submucosal location and nonspecific imaging features, which can mimic other tumors, such as GISTs. In this context, immunohistochemical analysis (especially demonstration of strong S100 protein expression) plays a critical role in confirming the diagnosis [22,27]. In addition to the classic presentations, rare variants and hybrid forms have been identified. Some lesions display overlapping immunophenotypic features, co-expressing markers associated with both Schwann and perineurial differentiation, broadening the histopathological spectrum of these tumors. Furthermore, in patients with a history of other malignancies, distinguishing a new schwannoma from tumor recurrence can be particularly challenging, emphasizing the importance of long-term follow-up [23,24].

Complete surgical resection with clear margins remains the treatment of choice for colorectal schwannomas. This approach not only provides a definitive histopathological diagnosis but is also associated with favorable clinical outcomes [25,28].

## 2. Case Report

### 2.1. Case Presentation

A 57-year-old woman with a significant medical history of total hysterectomy and bilateral adnexectomy for infiltrative serous ovarian carcinoma (3 years earlier) was referred to the Institute of Oncology “Prof. Dr. Ion Chirircuță” Cluj-Napoca for evaluation of a suspected tumor in the sigmoid colon. The patient had undergone successful treatment for ovarian carcinoma and was in remission. The new diagnosis was made based on the presence of a suspicious lesion observed during routine follow-up imaging.

### 2.2. Diagnosis and Treatment

**Figure 1:**
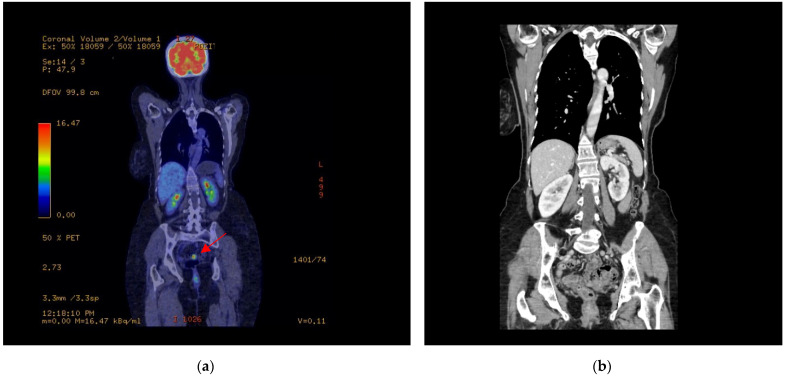
(**a**) Coronal fused PET/CT image demonstrating metabolic activity throughout the body. Physiological uptake is observed in the brain, kidneys, and bladder. A focal area of increased FDG uptake is identified in the lower abdomen, corresponding to the sigmoid colon (arrow), consistent with a metabolically active lesion; (**b**) Coronal contrast-enhanced CT scan of the thoracoabdominal region. The sigmoid colon is visible in the lower abdomen, with no obvious signs of obstruction or perforation. No lymphadenopathy or distant organ lesions are noted.

A PET-CT scan was performed on August 22 in order to assess the metabolic activity of the lesion (Figure 1). The scan confirmed the presence of a metabolically active lesion, which was located in the sigmoid colon, corresponding to the mass which had previously been identified on MRI. No additional areas of abnormal metabolic activity were observed in other abdominal organs, ruling out distant metastases.

**Figure 2:**
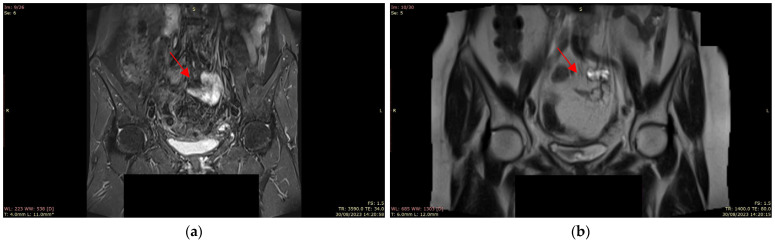
(**a**) Coronal post-contrast T1-weighted MRI showing a 20 mm enhancing submucosal lesion in the sigmoid colon (arrow), with well-defined margins and no adjacent invasion; (**b**) Coronal T2-weighted pelvic MRI showing a well-defined submucosal lesion in the anterior sigmoid colon wall (arrow), measuring approximately 17–22 mm, with intermediate-to-high signal intensity and no signs of adjacent invasion.

An abdominal and pelvic MRI (30 August) revealed a lesion in the sigmoid colon, approximately 17–22 mm in size. The lesion was hyperintense on T2-weighted images with limited diffusion and contrast enhancement, suggesting a neoplastic process. The tumor had well-defined margins and appeared to be attached to the anterior wall of the sigmoid colon. No evidence of distant metastasis or lymphadenopathy was identified (Figure 2).

Colonoscopy (performed on 15 September) revealed multiple polyps in the ascending, descending, and sigmoid colon, which were excised as benign conditions. A submucosal lesion, approximately 20 mm in diameter, was noted in the sigmoid colon. The mucosa overlying the lesion appeared normal, but the submucosal location raised the suspicion of a mesenchymal tumor. Biopsy specimens were obtained for histopathological evaluation.

**Figure 3:**
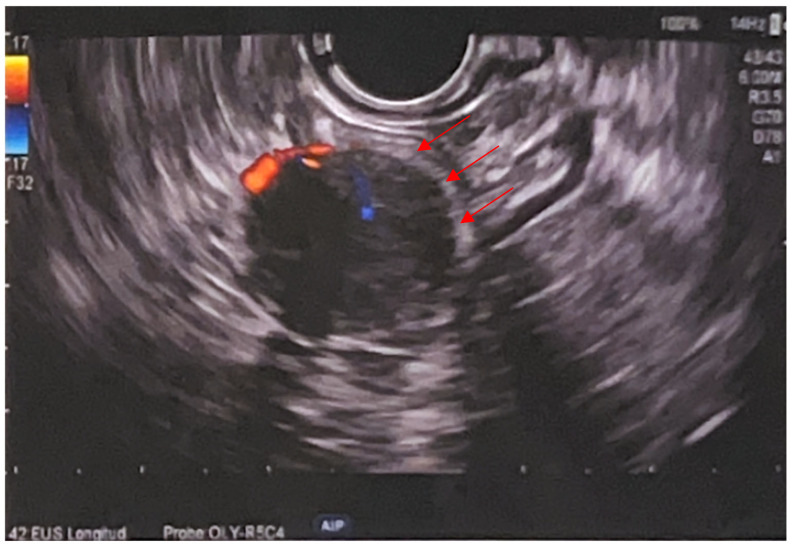
Endoscopic ultrasound (EUS) showing a well-defined, hypoechoic, vascularized submucosal lesion (20 × 16 mm) arising from the muscularis propria of the sigmoid colon (arrows).

Endoscopic ultrasound (EUS), performed on 26 September, revealed a 20 × 16 mm submucosal, well-defined, round, hypoechoic and heterogeneous lesion at 25 cm from the anal verge, arising from the muscularis propria. The lesion showed an internal Doppler signal and was firm on elastography (SR 2). Two passes of fine-needle biopsy (22G) were performed, yielding scant material due to difficult positioning. No perisigmoid lymphadenopathy was noted (Figure 3).

Biopsy samples obtained during colonoscopy (taken from submucosal lesions, not from polyps) were further subjected to cytological examination. The analysis revealed (2 October) the presence of fus-shaped cells with oval nuclei, fine granulated chromatin, and a small nucleolus, which are characteristic of GIST. Cytoplasm was moderately abundant and eosinophilic, further supporting the diagnosis of a GIST. The findings were consistent with histological features commonly found in GIST, confirming the diagnosis of GIST in the sigmoid colon.

On 31 October, the patient underwent a low anterior resection (Dixon Procedure) with total mesorectal excision. The procedure was performed under general anesthesia through a median supraumbilical-pubic incision. A low mechanical colorectal anastomosis was performed. Postoperative recovery was uneventful, with resumption of bowel movements on postoperative day 5. No signs of infection or complications were observed.

**Figure 4:**
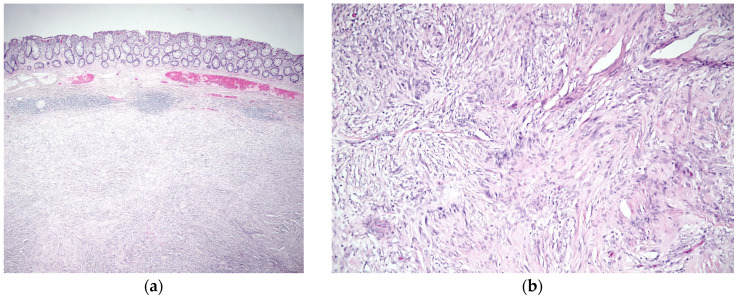
The resected specimens were fixed with 10% formalin fixative and 95% ethanol fixative, dehydrated, embedded in wax, sectioned, and stained with hematoxylin and eosin (Tissue-Tek Prisma). (**a**) 40×—Schwannoma, overview. Low-power view shows a well-circumscribed submucosal lesion beneath the colonic mucosa, composed of intersecting bundles of spindle-shaped cells exhibiting a storiform arrangement. The overlying mucosa is intact, and there is no evidence of ulceration or invasion into adjacent layers. The lesion demonstrates a fascicular architecture, characteristic of a mesenchymal neoplasm such as schwannoma; (**b**) 200×—Schwannoma, cellular morphology. At higher magnification, the lesion is composed of spindle cells with elongated, wavy nuclei arranged in interlacing fascicles. Nuclear palisading is focally observed, along with areas of collagen deposition between the cellular bundles.

**Figure 5:**
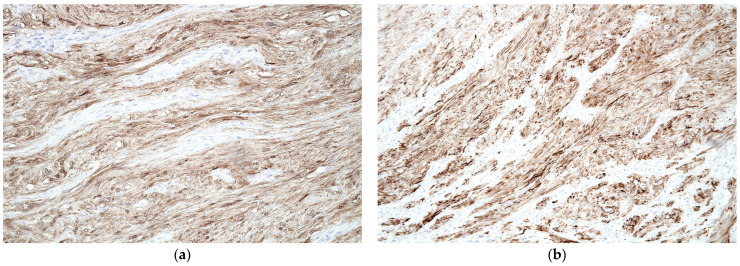
Positive Immunohistochemical Markers Supporting Schwannian Differentiation (S100, GFAP). (**a**) S100, 400×—Schwannoma, strong immunoreactivity. High-power view showing diffuse and intense nuclear and cytoplasmic positivity for S100 protein in the spindle cells. This strong, uniform staining pattern is highly characteristic of schwannoma, confirming the neural crest origin of the tumor cells. S100 is considered the most sensitive and specific immunohistochemical marker for schwannomas.; (**b**) GFAP, 200×—Schwannoma, positive immunostaining. Intermediate magnification image demonstrating cytoplasmic positivity for GFAP (glial fibrillary acidic protein) in spindle cells. GFAP expression further supports the glial/neuroectodermal differentiation of the lesion. While not as specific as S100, it provides additional evidence for diagnosis of schwannoma.

Histopathological analysis of the resected specimen confirmed the diagnosis of a schwannoma rather than a GIST. Several macroscopic and microscopic findings characterized the lesion. The surgical specimen corresponded to a recto-sigmoid resection measuring 10 cm in length and 5.3 cm in diameter. A 2.2 cm nodular tumor mass, firm in consistency, was observed, located 3 cm from the distal resection margin and 5 cm from the proximal resection margin. The cut surface of the tumor was yellowish, well-demarcated, and free of mucosal ulceration. Histological sections showed a proliferation of spindle cells with indistinct cytoplasmic borders, pale eosinophilic cytoplasm, and oval-shaped nuclei with fine granular chromatin. The tumor had a storiform arrangement with areas of nuclear palisade and focal hyalinization. There was no evidence of necrosis or significant mitotic activity, with a mitotic index of 1 mitosis per 10 high-power fields (Figure 4). Immunohistochemistry demonstrated strong positivity for S100 and SOX10, with negative staining for c-KIT (CD117), DOG1, and CD34, excluding GIST. The proliferation index (Ki67) was 10% (Figure 5). No tumor involvement was observed in the proximal and distal surgical margins or in the 12 lymph nodes examined. Omental sections showed areas of fibrosis, microhemorrhages, and mixed inflammatory infiltrates, with no evidence of malignancy.

### 2.3. Case Particularities and Key Features

Schwannomas of the gastrointestinal tract are rare, especially in the colon. They are usually asymptomatic or present with nonspecific gastrointestinal symptoms. Unlike GIST, which arises from the interstitial cells of Cajal, schwannomas arise from the nerve sheath and have distinct histopathological and immunohistochemical profiles. MRI and PET-CT scans are useful in detecting and characterizing these tumors, but definitive diagnosis is made by histological and immunohistochemical analyses. Schwannomas consistently express S100 and SOX10, while being negative for CD117, DOG1, and CD34, distinguishing them from GIST. Complete surgical excision remains the gold standard for treatment, and the prognosis is generally excellent, given the benign nature of these tumors. Unlike GIST (which may require adjuvant tyrosine kinase inhibitors such as imatinib), schwannomas do not usually require additional oncological treatment after resection.

This case highlights the diagnostic challenge of distinguishing schwannomas from other mesenchymal tumors, particularly GISTs. While schwannomas are exceedingly rare in the colon, their clinical and imaging characteristics can mimic those of GISTs, which are more commonly encountered in this location. In this instance, initial imaging findings, including the PET-CT and MRI, suggested a tumor consistent with a GIST, showing increased metabolic activity and features typical of a submucosal lesion. One of the challenges in this case was the limited cellular material obtained through EUS-guided FNA, which led to initial diagnostic uncertainty. Given the submucosal location and differential diagnosis that included GIST, this highlights the potential benefit of using larger-core biopsy needles or considering upfront molecular profiling in selected cases. Such approaches may improve diagnostic yield and help guide appropriate therapeutic strategies early in the diagnostic process. However, the final diagnosis was established through histopathological and immunohistochemical testing, which revealed distinct features of schwannoma, including a storiform pattern of spindle cells with characteristic eosinophilic cytoplasm. Additionally, immunohistochemistry revealed strong positivity for S100 and SOX10, which is typical of schwannomas, and negative staining for markers like c-KIT, DOG1, and CD34, which are associated with GISTs.

This case underscores the significance of accurate histopathological and immunohistochemical diagnosis in differentiating between these two types of mesenchymal tumors. Although the imaging characteristics might initially suggest a GIST, only careful microscopic examination and marker analysis can confirm a schwannoma diagnosis. Also, even if schwannomas typically express S100 and SOX10, immunohistochemical evaluation was not performed on the initial biopsy due to the limited material obtained via EUS-FNA. This led to diagnostic uncertainty, as morphological features alone were insufficient for a definitive diagnosis. This underscores a common pitfall in managing submucosal colonic tumors: namely, the low diagnostic yield of small-caliber biopsies. Adequate tissue sampling, preferably through core needle biopsy, is essential to allow comprehensive immunohistochemical analysis and avoid diagnostic delays. The successful surgical resection of the tumor, without the need for additional oncological therapies such as tyrosine kinase inhibitors, highlights the importance of precise diagnosis, as it directly impacts treatment decisions. Moreover, complete surgical excision of schwannomas typically leads to an excellent prognosis, reinforcing the benign nature of these tumors compared to the more aggressive GISTs.

Ultimately, the case emphasizes the importance of considering schwannomas in the differential diagnosis of submucosal lesions in the colon, particularly when imaging findings are inconclusive or suggest the possibility of a GIST. Accurate diagnosis, achieved through comprehensive histopathological and immunohistochemical evaluation, is crucial in ensuring appropriate treatment and avoiding unnecessary interventions, such as the administration of imatinib, which is not required for schwannomas. The presented case also illustrates the need for a multidisciplinary approach, combining the expertise of pathologists, radiologists, and surgeons, to ensure the best possible patient outcome.

## 3. Materials and Methods

### 3.1. Search Strategy

A comprehensive literature search was performed using the PubMed database to identify studies related to GISTs and schwannomas, specifically located in the sigmoid colon. The search queries used were “GIST” AND “Sigmoid colon”, respectively “Schwannoma” AND “Sigmoid colon”, performed on 22 February 2025. The first selection initially yielded 46 articles, while the second identified 35 articles. Articles were included if they focused on tumors of the sigmoid colon or colorectal region and included case reports, case series, or case reports accompanied by a literature review. Studies that focused on other pathologies or tumors located in anatomical sites other than the sigmoid colon were excluded from the review. After screening of titles and abstracts, 20 articles from the first selection and 15 articles from the second selection met the inclusion criteria and were used for further analysis.

### 3.2. Data Extraction, Synthesis, and Data Analysis

The information extracted included study design, patient demographics, clinical presentation, imaging findings, histopathological features, treatment strategies, and clinical outcomes. A qualitative analysis of the collected data was performed to identify trends and gaps in the current literature. Key findings were organized by diagnostic techniques, therapeutic interventions, and prognostic factors. This approach allowed for a comprehensive overview of the clinical and pathological spectrum of GIST and sigmoid colon schwannomas, as well as the challenges associated with their management.

## 4. Results

The present review synthesizes findings from a series of case reports and studies focusing on gastrointestinal stromal tumors and related mesenchymal neoplasms of the sigmoid colon and rectum. The reviewed literature illustrates the wide spectrum of clinical presentations, imaging characteristics, histopathological findings, and management strategies (Table 1). In many cases, the diagnosis was challenging due to overlapping features with other neoplasms, emphasizing the need for a comprehensive diagnostic workup.

### 4.1. Colorectal Gastrointestinal Stromal Tumors

In a case report by Imataki et al., a 44-year-old man with abdominal pain and melena was diagnosed with a gastrointestinal stromal tumor, causing severe anemia. Imaging revealed a spastic, S-shaped sigmoid colon, indicating spastic intestinal paralysis due to active internal bleeding. A 2.5 cm intraluminal polypoid tumor was identified on CT scan, and after partial resection, the diagnosis of GIST was confirmed. This case highlights the need to consider GIST in patients with an S-shaped sigmoid colon on imaging, especially with gastrointestinal bleeding [1].

In a case report by Shintaku et al., a 68-year-old Japanese man with sudden-onset abdominal pain was clinically diagnosed with gastrointestinal perforation and underwent emergency surgery. Intraoperatively, a ruptured diverticulum was identified in the sigmoid colon without an obvious tumor. Histological examination revealed diffuse proliferation of spindle cells infiltrating the muscularis propria, with immunohistochemical positivity for KIT, DOG1, and CD34. Mutational analysis revealed a heterozygous deletion in exon 11 of the c-kit gene. This case highlights the importance of recognizing planar GISTs, which can mimic other conditions, including perforated diverticulitis [2].

In a case reported by Nomura et al., a 67-year-old man was evaluated for fecal occult blood, which led to the detection of a 25 mm pedunculated polyp in the sigmoid colon. Endoscopic mucosal resection confirmed a pT1b adenocarcinoma with minimal vascular invasion. Subsequent laparoscopic resection revealed hyperplastic spindle cells in the muscularis layer at the scar site, positive for c-kit, CD34, and DOG1. This case highlights the rare coexistence of early sigmoid colon cancer and GIST [3].

In a case reported by Ueno et al., a 72-year-old man underwent emergency surgery for stercoral perforation of the sigmoid colon. Pathological examination revealed a proliferation of spindle cells replacing the muscularis propria without a distinct mass. The cells were positive for KIT and CD34, with a mutation in the c-kit gene confirming the diagnosis of planar GIST. The perforation was probably due to coprostasis caused by abnormal peristalsis at the site of the lesion. This case, together with two similar reports, suggests that planar GISTs should be considered in cases of idiopathic colonic perforation [4].

In a case reported by Sumi et al., an 80-year-old man underwent a Hartmann procedure for rectal cancer, during which a 9 mm submucosal tumor was found incidentally in the proximal sigmoid colon. Histopathological analysis revealed a proliferation of spindle cells with eosinophilic globules, consistent with a low-risk GIST containing skeinoid fibers. The tumor was positive for CD34, CD117, and vimentin, with a low MIB-1 index. Skeinoid fibers are rare in colonic GISTs, with only nine cases reported, highlighting their unusual occurrence in the large intestine [5].

Thway et al. reported a rare case of intra-abdominal fibromatosis in a 44-year-old woman who presented 3 years after resection of a sigmoid mesocolon GIST treated with imatinib. A mass at the site of the ileocolic anastomosis was initially suspected as a recurrent GIST, but histological and genetic analysis confirmed desmoid fibromatosis with a mutation in the CTNNB1 (β-catenin) gene. This case highlights the diagnostic challenge, as imaging alone cannot reliably differentiate between recurrent GIST and fibromatosis, underscoring the need for histopathological evaluation for accurate management [6].

Ijeri et al. described a case of gastrointestinal stromal tumor, mimicking an ovarian tumor in a gynecologic oncology setting. The patient presented with an adnexal mass, initially suspected to be of ovarian origin. However, further evaluation and histopathological examination confirmed the diagnosis of GIST. This case highlights the importance of considering GIST in the differential diagnosis of pelvic masses, particularly when imaging and clinical findings suggest an ovarian neoplasm. Accurate diagnosis is crucial, as the management and prognosis of GIST differ significantly from ovarian tumors [7].

Kamionkowski et al. reported a case of solitary sigmoid perineurioma in a healthy 30-year-old man who presented with chronic constipation. Colonoscopy revealed a 3–4 mm sessile polyp in the sigmoid colon, which was histologically confirmed as a perineurioma. These rare benign fibroblastic polyps arise from peripheral nerve sheath cells and present spindle-shaped cells with ovoid nuclei in a spiral pattern. The differential diagnosis includes ganglioneuromas, schwannomas, neurofibromas, and gastrointestinal stromal tumors [8].

Kobayashi et al. reported a rare case of a 79-year-old woman with a gastrointestinal stromal tumor who presented with recurrent cerebral infarctions, initially mimicking ovarian cancer with Trousseau syndrome. A giant pelvic tumor was detected, and after surgery, the tumor was diagnosed as GIST based on histopathology, showing spindle cell proliferation and positive markers for c-kit, CD34, and DOG1. Despite aggressive surgical interventions, including total hysterectomy and sigmoid colon resection, the patient suffered recurrent strokes. The case highlights the challenges in diagnosing GIST preoperatively, especially when it mimics other malignancies [9].

Lee et al. described the case of a 47-year-old woman who presented with a large pelvic mass (30 cm) and elevated CA-125 levels, initially suggesting an ovarian malignancy. Following exploratory laparotomy, the mass was identified in the sigmoid colon and diagnosed as a gastrointestinal stromal tumor. This case highlights the rarity of GIST, which can present as pelvic masses, often leading to misdiagnosis as gynecological diseases. Preoperative diagnosis of GIST is challenging, and non-gynecological causes must be considered when evaluating atypical pelvic masses. Gynecologists should be aware of extraovarian pathologies in such cases [10].

Stemate et al. demonstrated the successful use of colonic capsule endoscopy (CCE) in the diagnosis of a sigmoid gastrointestinal stromal tumor after two failed attempts at colonoscopy. CCE revealed a submucosal mass, which led to surgical removal of the tumor. This case is significant because it presents the first documented example of colonic GIST identified by capsule endoscopy, highlighting the utility of CCE as a valuable diagnostic tool in cases where colonoscopy fails or is contraindicated [11].

The case of Yamashita et al. consists of a rare diffusely infiltrating GIST in the sigmoid colon that caused perforation in a 72-year-old woman. Despite being undetectable preoperatively, the tumor was identified by microscopic examination as a spindle cell lesion with a diffuse growth pattern, KIT-positive, and with a C-KIT gene mutation. The case highlights the importance of histological, immunohistochemical, and genetic analyses in the diagnosis of such lesions, which have been linked to colonic perforation [12].

Hwangbo et al. reported the first documented case of pneumoretroperitoneum caused by the spontaneous rupture of a sigmoid colon gastrointestinal stromal tumor. A 77-year-old woman presented with acute abdominal pain and hematochezia. Imaging revealed a 9.7 × 9.3 cm mass in the pelvic cavity with air in the retroperitoneum. Emergency surgery identified a ruptured GIST with localized peritonitis. Pathology confirmed that the tumor was composed primarily of round epithelioid cells, immunohistochemically positive for CD34 and negative for C-kit [13].

Matsui et al. reported two cases of ultrasound-guided fine needle aspiration of pelvic lesions through the proximal sigmoid colon using a novel convex-type EUS scope. Case 1 involved a 77-year-old woman with multiple enlarged lymph nodes diagnosed with diffuse large B-cell lymphoma. Case 2 involved a 60-year-old woman with a large mass in the left lower abdomen diagnosed as a gastrointestinal stromal tumor (GIST). The new convex-type EUS scope with a wide-angle optic allowed precise targeting of lesions, demonstrating EUS-FNA as a valuable technique for diagnosing challenging pelvic lesions [14].

Miyake et al. reported the case of a 51-year-old woman who developed a parasitic leiomyoma in the mesentery of the sigmoid colon six years after undergoing total laparoscopic hysterectomy with morcellation in the abdomen. The mass, measuring 21/12/15 cm, was initially suspected to be a leiomyoma or potentially a malignant tumor such as a sarcoma or gastrointestinal stromal tumor, due to its size and location. Upon resection, the tumor was confirmed to be a benign leiomyoma. The case highlights the importance of considering parasitic leiomyoma in the differential diagnosis of solid abdominal tumors after gynecologic surgeries such as myomectomy or hysterectomy [15].

Nakajima et al. reported the case of a 46-year-old man with locally advanced GIST who underwent neoadjuvant therapy with imatinib mesylate, which resulted in a 60% tumor reduction. This was followed by successful surgical resection, including removal of the bladder, prostate, and part of the sigmoid colon. The patient remained on imatinib for 39 months without recurrence, highlighting the efficacy of neoadjuvant therapy in facilitating surgery for advanced GIST [16].

A study by Agaimy et al. describes two cases of sporadic segmental interstitial cell hyperplasia of Cajal (ICC), in which spindle cells replaced the intestinal wall, presenting as gastrointestinal stromal tumors. The first case involved a 59-year-old woman with a mass resembling a Meckel diverticulum, and the second a 66-year-old man with adenocarcinoma of the sigmoid colon. Both cases had somatic c-KIT mutations, distinguishing them from hereditary GIST syndromes. The study emphasizes the importance of differentiating sporadic ICC hyperplasia from hereditary GIST-related conditions [17].

Palaghia et al. reported the case of a 61-year-old male patient with a gastrointestinal stromal tumor, originating in the sigmoid colon that metastasized to the liver. The patient initially presented with vague, nonspecific symptoms, with a CT scan revealing the tumor origin and liver metastases. Surgery involved en bloc resection, and the patient was treated with Imatinib for the liver metastasis. Histological analysis showed a proliferation of spindle cells positive for CD117 and CD34. Despite complete resection, liver metastases remain a significant factor for recurrence [18].

In their study, Abbas et al. reviewed 13 patients with gastrointestinal stromal tumors, including gastric, small bowel, and sigmoid cases. Symptoms ranged from hematemesis to bowel perforation, some of which were discovered incidentally during endoscopy. Diagnosis was based on clinical features and histopathology. Most tumors were completely resected (R0), with C-kit positivity in all cases. Complications included gastrointestinal bleeding, biliary gastritis, and wound infection, with one tumor recurrence in the ileum [19].

Pari et al. reported a case of a 46-year-old man with a gastrointestinal autonomic nerve tumor (GAN) originating in the sigmoid colon who underwent surgery. Eight months later, an omental recurrence occurred, requiring a second successful laparotomy. The patient remained disease-free after 21 months of follow-up. This is the first reported case of a GAN tumor of the large intestine [20].

### 4.2. Colorectal Schwannomas

A case report by Kim et al. describes a 66-year-old woman with an asymptomatic schwannoma of the sigmoid colon discovered during a screening colonoscopy. A submucosal tumor was found, and although the biopsy was inconclusive, imaging showed a well-defined mass. After laparoscopic resection, the schwannoma was diagnosed based on S-100 protein positivity. Surgical margins were negative, and no metastases were found. Colonic schwannomas are rare, usually benign, and are often discovered incidentally. Surgical resection is the treatment of choice [21].

In a case reported by de Armas Conde et al., an 87-year-old woman with epigastric pain and dyspepsia was found to have a sigmoid neoplasm on CT and colonoscopy. The lesion, difficult to diagnose because of its submucosal location and nonspecific imaging features, was ultimately resected by laparoscopic sigmoidectomy. Histopathological analysis revealed a schwannoma, with immunohistochemistry showing S-100 positivity and negative staining for C-KIT, CD34, actin, and desmin—key findings that helped differentiate it from other mesenchymal tumors, such as GIST or leiomyomas [22].

Another study by Agaimy et al. describes two cases of schwannoma-perineurioma hybrid in the gastrointestinal tract, encountered in the gastric antrum and appendix. The tumors showed features of both schwannomas and perineuriomas, co-expressing S100, CD34, and perineurial cell markers. The findings suggest that these tumors are distinct from classic gastrointestinal schwannomas [23].

Suzuki et al. discussed the case of a 51-year-old man with a history of pancreatic neuroendocrine tumor (PNET) who developed a sigmoid schwannoma 13 years after surgery. MRI raised concerns about peritoneal dissemination, but laparoscopic resection revealed the schwannoma. The diagnosis was confirmed post-surgically, and the patient recovered. This case highlights the challenge of distinguishing schwannomas from recurrent PNET and the importance of long-term follow-up [24].

Çakır et al. described the case of a 79-year-old woman who presented with rectal bleeding and constipation. Endoscopy revealed a mass obstructing the sigmoid colon, and biopsies suggested a gastrointestinal tumor. After preoperative evaluation, she underwent sigmoid resection. Histopathology confirmed sigmoid schwannoma. The patient recovered uneventfully and was discharged on the fifth postoperative day. This case highlights the successful treatment of sigmoid schwannoma with total resection [25].

Selntigia et al. reported the case of a 46-year-old woman who presented with secondary amenorrhea, intermittent pelvic pain, and constipation. During a gynecological examination, a transvaginal ultrasound identified a solid mass in the sigmoid-rectal area. Colonoscopy and endoscopic submucosal dissection revealed a mass, which was resected en bloc. Immunohistochemical analysis showed strong S100 positivity in the tumor cells, confirming a benign sigmoid schwannoma. This case highlights the importance of a thorough pelvic examination and early diagnosis, especially in asymptomatic patients, to avoid delays in treatment [26].

Zainaldeen et al. reported the case of a 64-year-old man with a history of varicose veins, hypertension, and hyperlipidemia who presented with severe abdominal pain, nausea, and vomiting. CT and MRI revealed a 5 cm mass in the rectum, suggesting a gastrointestinal stromal tumor. A biopsy showed nonspecific inflammation, and subsequent laparotomy revealed a 10 cm exophytic schwannoma in the sigmoid colon. Histopathology confirmed the diagnosis. Schwannomas of the colon are rare, usually benign, and often asymptomatic, although they may cause abdominal discomfort. Surgical resection with clear margins is the treatment of choice, and the prognosis is generally favorable [27].

Nonose et al. reported the case of a 71-year-old woman with rectal bleeding, pain, and tenesmus for 4 months who was diagnosed with a 2.8 cm submucosal lesion in the sigmoid colon. Histopathology suggested a mesenchymal origin, and immunohistochemistry confirmed a benign primary schwannoma with strong S-100 expression. Because of compromised resection margins, colon resection was performed via videolaparoscopy. The patient recovered uneventfully and remained recurrence-free 15 months later [28].

In their work, Trivedi et al. present the case of a 61-year-old man who was found to have a 12 mm polyp in the sigmoid colon, diagnosed as a microcystic/reticular schwannoma, a rare variant more common in the large intestine. Histology showed vacuolated cells in a myxoid stroma, with no malignancy features. Immunohistochemistry was positive for S100, and negative for cytokeratin and CD117. This variant should be considered in the differential diagnosis of signet ring cell adenocarcinoma or myxoid GIST [29].

A rare case described by Vasilakaki et al. consists of a 68-year-old man with rectal bleeding who was diagnosed with two tumors: a 4.2 cm adenocarcinoma in the sigmoid colon and a 4 cm ancient schwannoma in the ascending colon. Both were identified by colonoscopy, and an ileo-hemicolectomy was performed. Histologically, the schwannoma showed degenerative changes and was positive for S100, while the adenocarcinoma was moderately differentiated. This rare case of synchronous colonic schwannoma and adenocarcinoma has not been widely reported in the literature [30].

Another case discussed by Sasatomi et al. is of a 68-year-old woman with hematemesis who was diagnosed with a submucosal tumor in the sigmoid colon. Tumor markers were normal, and she underwent sigmoid colectomy, revealing a yellowish tumor with necrosis, measuring 4.7 × 3.5 × 3.0 cm. Histology showed spindle cells with palisade nuclei and nuclear pleomorphism. Immunohistochemistry confirmed the tumor as a schwannoma, positive for S-100 protein and negative for actin staining. This case details a rare schwannoma of the sigmoid colon [31].

Horio et al. reported the case of a 66-year-old man with positive fecal occult blood who had a 3.0 cm hemispherical lesion in the sigmoid colon, identified by barium enema and colonoscopy. The tumor was surgically removed because of concerns about malignancy. Histology showed palisade spindle cells, and immunohistochemistry was positive for S-100 protein and vimentin, confirming a diagnosis of schwannoma [32].

Emanuel et al. described the case of a 48-year-old man who presented with constipation, hematochezia, and weight loss. Colonoscopy and CT revealed an obstructive colonic mass causing intussusception and pneumatosis, requiring emergency left hemicolectomy. Overall, the mass was 4.9 cm, extending through the muscularis propria. Histology showed spindle cells in a fibromyxoid stroma, with a spiral or storiform pattern, and bipolar cytoplasmic processes. Immunohistochemistry was positive for S-100, CD34, vimentin, and EMA, and negative for other markers. Electron microscopy showed features of both perineurioma and schwannoma. The tumor was diagnosed as a benign perineurioma-schwannoma hybrid, the first reported case in the colon [33].

Ahn et al. reported a rare case of sporadic colonic neurofibroma in a 26-year-old woman with a 4 cm sigmoid polypoid mass. Histology showed spindle cells, fibroblasts, and collagen fibers, with myxoid areas. Immunohistochemistry revealed positivity for CD34 and S-100, but negativity for c-Kit, DOG-1, and smooth muscle actin, confirming neurofibroma. Due to its rarity and expression of CD34, it may be misdiagnosed as GIST, emphasizing the need for accurate recognition to avoid unnecessary treatment [34].

Yang et al. described 10 cases of hybrid schwannoma/perineurioma, a rare benign nerve sheath tumor, in adult patients (age range 27–81 years, median 35), mostly female (8/10). Seven tumors were subcutaneous (trunk, extremities, neck, labium majus), and three were submucosal (nasal cavity, sigmoid colon, rectum). Histology showed a mixture of thick and thin spindle cells in storiform, lamellar, or fascicular patterns. Immunohistochemistry confirmed Schwann (S100+) and perineural (EMA, claudin-1, CD34) differentiation. Recognition of its different locations and characteristics aids in diagnosis [35].

## 5. Discussion

### 5.1. Integration of a Multimodal Diagnostic Approach

Our case highlights the critical importance of a comprehensive diagnostic strategy in the evaluation of mesenchymal tumors in atypical locations, such as the sigmoid colon. Initial suspicion of a gastrointestinal stromal tumor was based on imaging findings, including PET-CT assessment of metabolic activity and MRI features such as T2 hyperintensity, restricted diffusion, and contrast enhancement. These findings led to the presumption of a GIST, given the well-defined, submucosal appearance of the lesion. Colonoscopic evaluation with targeted biopsy revealed a fusiform morphology, reinforcing the initial diagnostic impression. However, definitive diagnosis requires histopathological and immunohistochemical analyses, highlighting the need for a multimodal approach in such cases.

### 5.2. Surgical Management and Postoperative Outcomes

In the presented case, the decision to perform a low anterior resection (Dixon procedure) with complete mesorectal excision was based on the localized nature of the lesion and the absence of metastases. Rigorous preoperative preparation, including mechanical bowel debridement, allowed for a meticulously planned surgery that achieved an R0 resection. Postoperative recovery was uneventful, with rapid recovery and no major complications. However, the final histopathological examination revealed that the lesion was not a GIST, but rather a schwannoma. This reinforces the importance of histological confirmation before proceeding with targeted therapies, as treatment strategies differ significantly between these tumor types.

### 5.3. Correlation with Current Literature

A review of the literature reveals a wide spectrum of clinical presentations for mesenchymal tumors of the sigmoid colon, ranging from incidental findings to emergent cases involving perforation or significant hemorrhage. Initial diagnostic confusion between GIST and schwannomas is well documented due to the submucosal location and similar imaging features. However, definitive differentiation relies on immunohistochemical markers. GISTs are consistently positive for CD117, DOG1, and CD34, whereas schwannomas, as in our case, express S100 and SOX10 while being negative for CD117 and DOG1.

Furthermore, minimally invasive diagnostic techniques (including colonoscopy and ultrasound-guided fine-needle aspiration) have emerged as valuable tools for obtaining definitive histopathological confirmation, thus guiding appropriate therapeutic strategies. Unlike GIST, which may require adjuvant therapy with imatinib mesylate, schwannomas usually require only surgical excision, with an excellent prognosis and minimal risk of recurrence.

### 5.4. Challenges and Future Perspectives

Despite the generally favorable outcomes associated with complete surgical resection, distinguishing between the various mesenchymal tumors of the gastrointestinal tract remains a significant diagnostic challenge. The case presented here highlights the limitations of imaging alone in differentiating GIST from other spindle cell neoplasms, reinforcing the need for a multidisciplinary approach that includes expertise in pathology, radiology, and gastroenterology.

Future research should focus on refining noninvasive diagnostic tools, including the role of molecular markers and artificial intelligence in distinguishing between the various mesenchymal tumors. In addition, the integration of novel imaging techniques and the expanded use of liquid biopsy and molecular profiling could further improve early and accurate tumor classification. This case serves as a reminder that even when imaging findings strongly suggest a particular diagnosis, histopathological confirmation remains essential in guiding optimal patient management.

## 6. Conclusions

Collectively, the heterogeneity of sigmoid colon mesenchymal tumors is highlighted in the current literature. Integration of advanced imaging, histopathological evaluation, and targeted therapies plays a critical role in making accurate diagnoses and optimizing patient outcomes. The findings support a multidisciplinary approach, especially in cases where clinical presentations are atypical or overlap with other pelvic pathologies. In the reviewed literature, imaging modalities such as MRI, PET-CT, colonoscopy, and EUS-FNA consistently emerged as critical tools in identifying and characterizing sigmoid colon lesions. Differential diagnosis frequently requires histopathological and immunohistochemical confirmation to distinguish GIST from other submucosal tumors or benign mimics. Surgical management strategies varied significantly depending on tumor presentation. Elective resections, often preceded by neoadjuvant imatinib mesylate therapy, achieved favorable outcomes, whereas emergency interventions were required in cases of perforation or hemorrhage.

The importance of a multidisciplinary approach was evident, especially in cases where the clinical presentation mimicked other pelvic pathologies, such as ovarian neoplasms or perineuriomas. Finally, the literature emphasizes that despite generally favorable outcomes after surgery, the risk of recurrence and metastasis remains a clinical challenge. Long-term follow-up and the potential for targeted therapies are critical considerations to optimize patient care and improve prognostic outcomes.

## Figures and Tables

**Table 1:** Comparative Table: GISTs versus Colorectal Schwannomas.

Feature	GISTs	Colorectal Schwannomas	Sources
**Frequency**	Rare in the colon; more common in the stomach and small intestine	Very rare; 2–6% of all GI schwannomas; 12% occur in small and large intestines	[1,2,3,22]
**Preferred Location**	Sigmoid colon	Submucosal lesions in the colon and rectum	[1,21]
**Clinical Presentation**	Ranges from asymptomatic to GI bleeding, perforation, or mimicking pelvic pathologies	Usually incidental; may cause abdominal pain, rectal bleeding, constipation	[1,2,4,22]
**Differential Diagnosis**	May mimic ovarian tumors or peripheral nerve sheath tumors	Can be confused with GISTs or other mesenchymal tumors	[7,9,10,27]
**Diagnostic Imaging** **Techniques**	MRI, PET-CT, colonoscopy, EUS-FNA	Nonspecific imaging; colonoscopy; difficult to diagnose preoperatively due to submucosal location	[1,2,5,22]
**Histopathology**	Spindle cell morphology; immunopositive for CD117, DOG1, CD34	Strong S100 protein positivity; sometimes co-expressing CD34; hybrid schwannoma-perineurioma reported	[3,6,7,8,21,23]
**Rare Variants**	GAN tumors, segmental ICC hyperplasia, spontaneous perforation, pneumoretroperitoneum	Microcystic or reticular forms; hybrid lesions expressing both Schwann and perineurial markers	[12,13,14,15,16,17,23]
**Main Treatment**	Complete surgical resection (R0); targeted therapy (imatinib) in selected cases	Complete surgical excision with clear margins; typically curative	[16,25,28]
**Role of Targeted Therapy**	Imatinib used in neoadjuvant/adjuvant settings in locally advanced or recurrent cases	Not typically used	[16]
**Need for Long-term** **Follow-up**	Yes, due to risk of metastasis or recurrence	Yes, especially in hybrid forms or patients with history of malignancy	[18,19,20,23,24]

## Data Availability

Data are available upon reasonable request.

## Abbreviations

The following abbreviations are used in this manuscript:

GIST	Gastrointestinal stromal tumors
MRI	Magnetic resonance imaging
CT	Computed tomography
PET-CT	Positron emission tomography-computed tomography
EUS	Endoscopic ultrasound
FNA	Fine-needle aspiration
EUS-FNA	Endoscopic ultrasound-guided fine-needle aspiration
CCE	Colonic capsule endoscopy
ICC	Interstitial cell hyperplasia of Cajal
GAN	Gastrointestinal autonomic nerve tumor
PNET	Pancreatic neuroendocrine tumor
